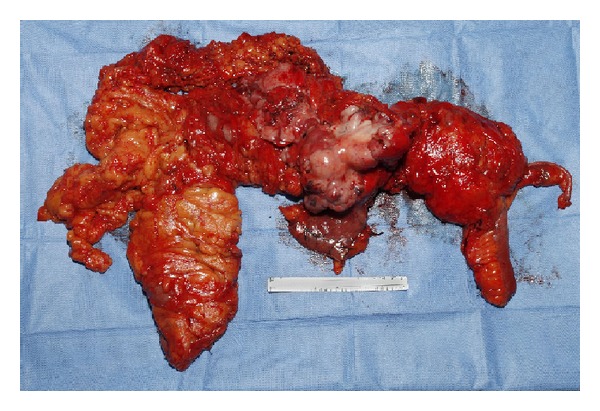# Traumatic Implantation: A Novel Aetiology in the Development of Peritoneal Mesothelioma

**DOI:** 10.1155/2013/389130

**Published:** 2013-12-26

**Authors:** Nicola Humphrys, Amy Downing, Luke Evans, Martin Sinclair

**Affiliations:** ^1^Emergency Medicine, Addenbrooke's Hospital, Cambridge, UK; ^2^Histopathology, East of England Deanery, Cambridge, UK; ^3^General Surgery, Norfolk & Norwich University Hospital, Norwich, UK; ^4^Ipswich Hospital, Suffolk, UK

## Abstract

Peritoneal mesothelioma is a rare intra-abdominal malignancy. Its aetiology has been thought to be due to either inhalation or ingestion of asbestos particles. We present a case of peritoneal mesothelioma developing as a result of a novel third route and the inoculation of fibres into the peritoneal cavity by penetrating trauma and direct transport. This case report highlights the important long term consequences of penetrating abdominal trauma and the need for vigilance in undertaking peritoneal toilet.

## 1. Introduction

Malignant mesothelioma of the peritoneum is a rare malignancy, but one that is increasing in incidence. Prognosis is poor and almost universally fatal. The untreated median survival rate is 6 months and even with the most aggressive multimodal therapy in the form of debulking or cytoreductive surgery and heated intraperitoneal chemotherapy five year survival ranges only from 29% to 59% [1, 2].

As treatment is associated with high morbidity and ultimate failure, a large amount of work is done to reduce exposure to asbestos and other nonasbestos mineral fibres such as Erionite which are implicated in the development of the disease. Typically the inhalation of fibres precedes the peritoneal inoculation and therefore it can be hoped that environmental health procedures on the handling of asbestos will start to have an effect on the incidence of peritoneal mesothelioma in the majority of cases [3]. This case would unfortunately not be effected by such measures due to the novel aetiology of inoculation of the fibres as described.

## 2. Case Presentation

A 68-year-old male presented to the surgical outpatient department after failure of medical management of his dyspeptic symptoms. His presenting symptoms were of worsening dyspepsia, anorexia, and a small degree of weight loss. His past medical history included mild ischaemic heart disease and a previous deep vein thrombosis. The patient also described having had a laparotomy more than 20 years previously.

Whilst working as a roofer, the patient had fallen from a height onto the spike of a garden parasol. The patient removed the impaling object himself and presented to the emergency department. An entry wound in the perineum was noted, washed, and closed and the patient was admitted for observation. After 48 hours with no signs of illness, he was discharged but represented two weeks later with peritonitis.

At laparotomy, an abscess cavity containing pieces of clothing in the right upper quadrant was noted; no GI organs were breached or resected.

**Figure 1:**
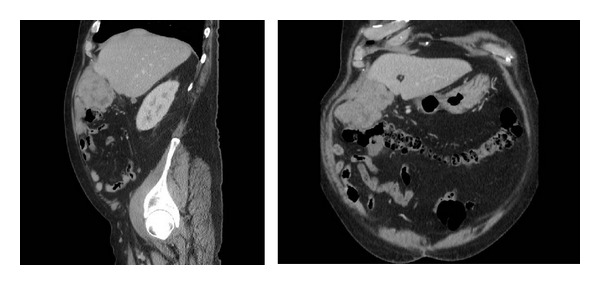


On examination in clinic 20 years later, he was found to have a midline laparotomy scar and a palpable, indistinct, and soft-feeling, nontender soft tissue mass in the right upper quadrant. CT (Figure 1) demonstrated this further and the patient subsequently underwent percutaneous core biopsy of the mass.

Biopsy showed that the specimen contained tissue infiltrated by cords of malignant and polygonal cells. Immunohistochemistry showed the cells stained positively for CK5, CK6, Calretinin, WT1, CK7, and CD9 with weakly focally positive staining for BerEP4 and NSE. H & E staining favoured a diagnosis of metastatic, poorly differentiated adenocarcinoma. An alternative diagnosis of desmoplastic small round cell tumour or mesothelioma could not be excluded, so further tests were done. The sample was stained negatively for desmin and the immunophenotype suggested mesothelioma differentiation. Correlation with the history and clinical findings concluded with most likely diagnosis being malignant mesothelioma.

**Figure 2:**
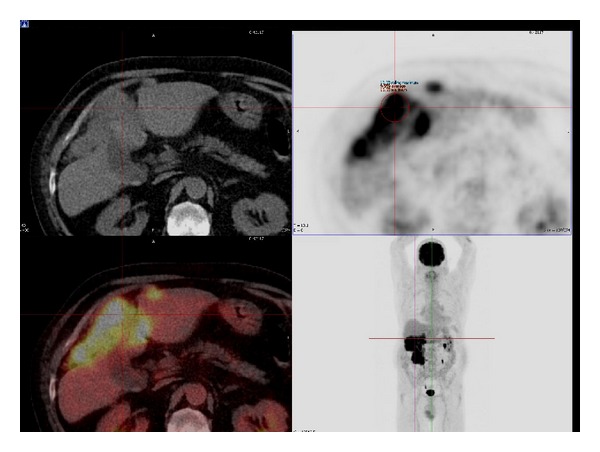


EGD, colonoscopy, and PET/CT (Figure 2) showed no other abnormality and the patient therefore proceeded to surgery.

At laparotomy, the patient underwent en bloc resection of the mass including distal stomach and right and transverse colon, Figure 3.

The postoperative histological analysis of the specimen confirmed the diagnosis of malignant mesothelioma. In the UK all such patients are discussed with one of the two national centres for peritoneal malignancy and in this case the decision was made for no further treatment.

The patient was followed up with regular CT scans. He remains alive at 19 months followup but unfortunately has recurrent disease in the abdomen.

## 3. Discussion

The main cause of mesothelioma is known to be exposure to asbestos fibres [4], but other nonasbestos mineral fibres and organic chemicals have been suggested as causative agents [5]. The patient's work as a roofer in the 1980s would have almost certainly involved some exposure to asbestos fibres. Asbestos fibres are known to remain on clothing [6] and therefore the passage of fragments of work-wear into the abdominal cavity poses a real route of inoculation.

The routes for the passage of mineral fibres to the peritoneal cavity are typically thought of in two ways. First the inhalational route where particles are trapped in respiratory secretions and transported via the lymphatics into the abdominal cavity. Second by ingestion of respiratory secretions in which the fibres have become trapped; there would subsequently need to be translocation from the gut lumen to the peritoneum. In the majority of cases caused by these routes of transport there will be evidence of pleural asbestos exposure. This patient had no evidence of pleural disease at time of presentation or, subsequently, making inhalational or ingestion unlikely.

The increased use of cross-sectional imaging in the modern management of penetrating trauma might nowadays have prevented the initial injury from being missed, but it is a warning tale for clinicians to remain vigilant in such cases, particularly when a patient self-presents seemingly untroubled by their wounds having previously removed the offending object themselves.

## Figures and Tables

**Figure 3:**